# Tenofovir in the treatment of hepatitis B virus infection after liver transplantation, a single center large population study 

**Published:** 2021

**Authors:** Fardad Ejtehadi, Mohammad Reza Pashaei, Alireza Shamsaeefar, Nasrin Motazedian, Gholam Reza Sivandzadeh, Ramin Niknam, Seyed Ali Malekhosseini, Kamran B Lankarni

**Affiliations:** 1 *Gastroenterohepatology Research Center, Shiraz University of Medical Sciences, Shiraz, Iran *; 2 *Department of Gastroenterology, Urmia University of Medical Sciences, Urmia, Iran*; 3 *Shiraz Organ Transplant Center, Shiraz University of Medical Sciences, Shiraz, Iran*; 4 *Shiraz Transplant Research Center, Shiraz University of Medical Sciences, Shiraz, Iran*; 5 *Health Policy Research Center, Institute of Health, Shiraz University of Medical Sciences, Shiraz, Iran*

**Keywords:** Hepatitis B, Liver transplantation, Therapeutics

## Abstract

**Aim:**

This study investigated the safety and efficacy of tenofovir disoproxil fumarate (TDF) compared with lamivudine (LAM) in the prevention of recurrent HBV infection after liver transplantation (LT).

**Background:**

Although the recurrence of hepatitis B virus after liver transplantation (LT) is now very uncommon with both nucleoside and nucleotide analogs represented with lamivudine and tenofovir disoproxil fumarate, respectively, few studies have compared the two classes.

**Methods:**

A total of 302 HBV-related post-transplant patients who received liver transplants from deceased donors were enrolled in this retrospective study from 2011 to 2015 in the Shiraz Organ Transplant Center, Iran. The demographic data, kidney function, recurrence, resistance rate, and acute rejections at 1-, 6-, and 12-month intervals and after 12 months were compared on TDF (n=209) and lamivudine (n=93) groups.

**Results:**

During a median follow-up period of 42.9 months, mean creatinine level was not significantly different between the two groups. Hepatitis B virus recurrence rate as well as acute graft rejection episode had no statistical difference in either group over the study period.

**Conclusion:**

Kidney function, creatinine level, disease recurrence, and acute graft rejection were comparable between tenofovir disoproxil fumarate and lamivudine in patients who received follow-up periods.

## Introduction

 The prevalence of hepatitis B virus (HBV) infection is about 400 million people in the world (1). In the general population of Iran, the prevalence rate of chronic HBV is estimated to be between 1.7% and 2.7% (2). HBV infection is one of the main reasons for liver cirrhosis and a major cause of hepatocellular carcinoma (HCC) globally (1). Despite many advances in the treatment of chronic HBV infection in recent years, the only effective treatment for end stage liver disease due to Hepatitis B virus is liver transplantation (LT). The recurrence rate of hepatitis B without antiviral prophylaxis after LT is 80%-100%, which results in a 50% mortality rate, in the two first years after transplantation (3).

Lamivudine (LAM), a nucleoside analog, has been effective in pre- and post-transplant phases by decreasing HBV replication and serum HBV DNA levels, yet drug resistance remains a major challenge in long-term use. More effective nucleotide analogs, such as entecavir and tenofovir disoproxil fumarate (TDF), have a much better profile regarding resistance. Indeed, no resistance has been reported with TDF; however, there are concerns regarding other side effects, especially with TDF (4).

In contrast to lamivudine, TDF maintains a very high genetic barrier to HBV resistance, even in patients infected with lamivudine resistant strains (5). 

Few studies have evaluated the efficacy and safety of TDF in preventing recurrent HBV infection following LT, and few have compared its safety and efficacy with LAM (5-9). 

This study investigated the safety and efficacy of TDF compared with LAM in the prevention of recurrent HBV infection after liver transplantation among patients in Shiraz, Iran. 

## Methods

The medical records of 302 patients who underwent LT for HBV-related end-stage liver disease in Shiraz Organ Transplant Center from 2011 to 2015 were retrospectively analyzed. Eligible patients were older than 18, and had acute fulminant or chronic HBV before LT. A written informed consent was obtained before transplantation from all patients.

**Antiviral therapy**

In the patients who received TDF or LAM before transplantation, the same drug was continued after transplantation. TDF was started when the patient did not receive any antiviral therapy before LT. In the patients who received lamivudine and showed drug resistance (confirmed by DNA PCR assay), LAM was changed to TDF. During the study period, tenofovir alafenamide fumarate was not available. 

**Prophylaxis with HBIG**

All patients received hepatitis B immunoglobulin (HBIG) at a dose of 2000 IU intramuscularly (IM) daily for one week after LT with control of anti-HBs antibody titer aiming to be above 500 IU/ml in the first six months. After the first week, HBIG doses were decreased step by step according to the anti-HBS antibody titer until finally 1000 IU was administered monthly or every 45 days for at least three years, with the aim of maintaining the anti-HBs antibody titer above 100 IU/m.

**Immunosuppression**

Mycophenolate mofetil tacrolimus and prednisolone were the mainstay of immunosuppression in transplanted patients. The mycophenolate mofetil dose was adjusted to 1000 mg every 12 h. Tacrolimus trough level was used to keep the medication at the therapeutic level, and prednisolone was given at 20 mg daily with dose reduction according to the transplanted liver function with the goal of discontinuation of the drug in month 3. All medications were monitored closely for possible side effects and interactions. Sirolimus, everolimus, and cyclosporine were used as alternative options.

**Statistical analysis**

Statistical package for the social sciences (SPSS) statistical software (Version 18.0; SPSS Inc. Chicago, IL, USA) was used As well as descriptive tests and chi square. A level of *p*<0.05 was considered statistically significant.

**Ethics**

The study protocol was approved by the Ethics Committee of Shiraz University of Medical Sciences (IR.SUMS.REC. 1394. S742).

## Results

A total number of 302 patients with documented HBV infection were enrolled in this study. Out of this number, 209 patients received TDF and 93 patients received LAM before LT and were maintained on the same drug after LT. The TDF group included 177 (85%) men and 32 (15%) women. The LAM group was comprised of 71 (76%) men and 22 (24%) women. The characteristics of the two groups are presented in Table 1. The two groups of patients did not differ significantly by gender (*p-*value=0.08). The median follow-up period was 42.9 months. 

During the follow-up period, creatinine levels in the TDF group were significantly lower at months one and six. After that, there was no statistically significant change in serum creatinine levels between the two groups. No significant difference was seen in the number of patients with a creatinine level higher than 2 mg/dL between the two groups during the follow-up period. The number of patients who had more than 50% increase in creatinine level at 6 and 12 months and after one year of follow up was not statistically different between the two groups (Table 2).

**Table 1:** Baseline characteristic of 302 patients treated with tenofovir or lamivudine

Variables	All patients	Tenofovir group	Lamivudine group
Total number of patients	302	209	93
Gender			
Male	248(82%)	177(85%)	71(76%)
Female	54 (18%)	32(15%)	22(24%)
Mean age	50.78± 10	49.89± 10.704	52.80± 8.380
Male	51.45± 9.8	50.71±10.277	53.31±8.273
Female	47± 11	45.34±12.002	51.14±8.703
HBS antigen Status			
HBS antigen + (n)	215	154	61
HBS antigen - (n)	87	55	32
Hbe Ag and Hbe Ab status			
Hbe Ag + (n)	42	29	13
Hbe Ag – (n)	260	180	80
Hbe Ab +ve (n)	146	101	45
Hbe Ab –ve (n)	156	108	48
Anti Hbc Ab IgG			
Positive (n)	201	142	59
Negative (n)	101	67	34
HBV DNA PCR			
Positive (n)	148	114	34
Negative (n)	154	95	59
Number of patients who received antiviral therapy before transplantation (n)	168	125	43
Median duration of antiviral therapy before transplantation(months)		12	18
History of antiviral related side effect(s) (n, %)	2 (1.2%)	2 (1.6%)	0 (0%)

**Table 2:** Comparison of lamivudine and tenofovir groups based on creatinine level, number of patients with creatinine level more than 2 mg/dL, and more than 50% increases in creatinine level

Variables	Lamivudine group	Tenofovir group	P. value
Creatinine level (mean ± SD; mg/dL)			
1month	1.39 ± 0.54	1.25 ±0 .39	0.016
6 months	1.27 ± 0.27	1.16 ± 0.31	0.008
12 months	1.19 ± 0 .23	1.19 ± 0.21	0.97
After 12 months	1.19 ± 0.20	1.19 ± 0.22	0.97
Number of patients with Creatinine level more than 2 mg/dL (n, %)			
1month	4 (4.3%)	12 (5.7%)	0.78 *
6 months	1 (1.3%)	1 (0.5%)	0.47 *
12 months	0 (0%)	2 (1%)	1*
After 12 months	0 (0%)	2 (1.1%)	1*
Number of patients with more than 50% increases in creatinine level (n, %)			
6 months	3 (4.1%)	6 (3%)	0.70*
12 months	1 (1.6%)	8 (3.2%)	0.68*
After 12 months	0 (0%)	8 (4.4%)	0.2*

* Fisher’s Exact Test

HBV recurrence rate after transplant which was detected by HBV PCR at 1-, 6-, and 12-month intervals and after 12 months was not significantly different between the groups (Table 3).

The rates of acute graft rejection in LAM and TDF groups were not statistically different (Table 4).

During the first one to twelve months of follow up, 5 and 13 patients died in the lamivudine and tenofovir group, respectively, which showed no statistically significant difference (*p-*value=1).

## Discussion

In this study, the safety and efficacy of TDF and LAM in patients who received LT for end-stage liver disease due to HBV were compared. There was no statistically significant difference between the two drugs regarding renal dysfunction, HBV recurrence, or occurrence of graft rejection.

While resistance to LAM is a major concern in patients on chronic use of this drug in a non-transplantation setting, the major concern for TDF 

chronic use is decline in renal function and bone density. This could be a major concern, as the use of other potentially nephrotoxic drugs in the transplanted patients such as calcineurin inhibitors is a major cause of late post-transplant morbidity and mortality (10). 

**Table 3:** HBV recurrence rate after transplant (positive DNA) in Lamivudine and Tenofovir groups during the follow up period

Variables	HBV Recurrence in 1 month (n, %)	HBV Recurrence in 6 months (n, %)	HBV Recurrence in 12 months (n, %)	HBV Recurrence after 12 months (n, %)
Lamivudine group	2 (2.1%)	1 (1.31%)	2 (3.2%)	3 (5.3%)
Tenofovir group	1 (0.5%)	4 (1.9%)	4 (2.1%)	5 (2.7%)
P. value	0.22*	0.1*	0.64*	0.34*

* Fisher’s Exact Test

**Table 4:** Acute rejection rate in Lamivudine and Tenofovir groups during the follow-up period

	Acute rejection (n, %) 1 month	Acute rejection (n, %) 6 months	Acute rejection (n, %) 12 months	Acute rejection (n, %) >12 months
Lamivudine group	5 (5.3%)	2 (2.6%)	4 (6.4%)	1 (1.8%)
Tenofovir group	18 (8.6%)	6 (2.9 %)	7 (3.7%)	3 (1.6%)
P. value	0.32	1*	0.47*	1*

* Fisher’s Exact Test

Unfortunately, bone densitometry was not done routinely for patients on LAM after LT in this study, so the effects of the two groups in this regard could not be compared; however, the data revealed that renal function deterioration after LT was not different between the two drugs. 

Other studies have been conducted on relatively small numbers of patients to evaluate the safety of TDF after LT. A study from Toronto, Canada, revealed no significant adverse events related to TDF or recurrent HBV infection in 24 patients who received LT for HBV-related disease during the median follow-up period of 29.1 months (7). However, there was no comparison to LAM. Furthermore, three of their patients died during follow up, one of them with chronic rejection. Another study from Germany compared the side effects of TDF with those of adefovir (which is currently not in use) post-LT and found no severe side effects (11). In a study from Turkey on 36 patients, half treated with LAM and half with TDF during the 36-month follow up post-LT, there was no difference in renal function, and the authors concluded that TDF therapy is safe in treating HBV-positive organ-transplanted patients.^6^ The same results were seen in one study on the effects of tenofovir monotherapy compared to entecavir (n=31) (12). Other studies had the same results in Spain on 22 patients (12) and in the United States (n=40) (13).

The main limitation of these studies was the small number of patients. A systematic review on the comparative efficacy of the newer nucleotide analogs with high genetic barriers [i.e. entecavir (ETV) or TDF] with LAM post-LT in a total of 519 patients who received LT for HBV-related liver disease from 11 studies found a lower recurrence rate with the former. Even in this systematic review, the total number of patients who received TDF was less than 100 (14). To the best of the authors’ knowledge, this report on the comparison of TDF and LAM is one of the largest series on this issue with 209 patients on TDF. 

This comparatively large study confirmed the results of previous smaller studies that patients who received TDF had no serious adverse renal events compared to those who received LAM. Another strength of the current report is the relatively longer follow up with a median follow-up period of 42.9 months. 

According to the results of the current study, the mean creatinine levels at intervals of 1, 6, and 12 months and after one year did not differ in the TDF group (n=209) compared with the LAM group (n=93). The numbers of patients in the two groups who developed at least 50% increase in creatinine level were compared and no difference was found. It must be emphasized that in cases of an increase in serum creatinine, first of all, drug side effects, especially those related to calcineurin inhibitors, should be considered, and the first step is to reduce the dose or even discontinue these medications. 

Recurrence of the primary disease is a major concern in post-transplant patients. LAM-resistant mutants developed in post–transplant recipients with prolonged use.^14^ Tenofovir has been considered as one of the alternative options for the treatment of patients with lamivudine-resistant HBV infection,(11, 15) with some concerns about the negative effects on kidney function (15). In one study, no HBV recurrence was reported among 14 of the post-transplant patients who received tenofovir (16). The results of our study on the large number of HBV patients who received tenofovir showed that in the post-transplant immunosuppression setting, recurrence of the disease may occur rarely. As shown in Table 3, however, there is no statistical difference in recurrence rate between the tenofovir and lamivudine groups. The emergence of tenofovir-resistant HBV is limited to case reports (17). According to the current results, the possibility of tenofovir resistance in post-transplant patients is much higher than what is expected in chronic hepatitis B; however, there was no statistical difference between the lamivudine group and the tenofovir group. The possible effect of immunosuppression on the emergence of tenofovir resistance is one explanation for this finding. Another explanation for this finding is the low compliance with tenofovir consumption in these patients. Unfortunately, gene study for possible mutation diagnosis was not available during the study periods.

The current results (Table 4) revealed that the acute rejection rate in both lamivudine and tenofovir groups has no statistically significant difference. The rejection rate after HBV liver transplant is lower than the other type of liver diseases (1), and the effect of lamivudine on 1-year and 5-year graft survival has been well established (18).There is limited data on the effect of tenofovir on short-term and long-term graft survival from the studies on a small number of patients (19). According to the current results on a large number of patients, the effect of tenofovir on acute rejection is equal to that of lamivudine, and this nucleotide analog has no negative effect compared with lamivudine in this regard.

The results of the current study on a large number of post-transplant patients indicate the satisfactory efficacy and safety of tenofovir in liver transplant. Tenofovir has an excellent result in preventing HBV infection recurrence in post-liver transplant patients. The drug is well tolerated without having a negative effect on renal function compared to lamivudine. The current study showed that tenofovir does not increase the rate of acute cellular rejection. Based on the results of this study, tenofovir resistance in post-transplant patients is much more frequent than what has been reported in non-transplanted chronic HBV patients. Evaluation of this finding, the reasons, and possible preventive measures needs further research.
